# Response of cord blood cells to environmental, hereditary and perinatal factors: A prospective birth cohort study

**DOI:** 10.1371/journal.pone.0200236

**Published:** 2018-07-06

**Authors:** Marco Patrick Lurà, Olga Gorlanova, Loretta Müller, Elena Proietti, Danielle Vienneau, Diana Reppucci, Rodoljub Pavlovic, Clemens Dahinden, Martin Röösli, Philipp Latzin, Urs Frey

**Affiliations:** 1 Division of Pulmonology, University Children’s Hospital Basel, University of Basel, Basel, Switzerland; 2 Swiss Tropical and Public Health Institute, University of Basel, Basel, Switzerland; 3 Institute of Immunology, Inselspital, University of Bern, Bern, Switzerland; 4 Division of Respiratory Medicine, Department of Paediatrics, Inselspital, University of Bern, Bern, Switzerland; University of North Carolina at Chapel Hill, UNITED STATES

## Abstract

**Background:**

Many studies investigating the impact of individual risk factors on cord blood immune cell counts may be biased given that cord blood composition is influenced by a multitude of factors. The aim of this study was to comprehensively investigate the relative impact of environmental, hereditary and perinatal factors on cord blood cells.

**Methods:**

In 295 neonates from the prospective Basel-Bern Infant Lung Development Cohort, we performed complete blood counts and fluorescence-activated cell sorting scans of umbilical cord blood. The associations between risk factors and cord blood cells were assessed using multivariable linear regressions.

**Results:**

The multivariable regression analysis showed that an increase per 10μg/m^3^ of the average nitrogen dioxide 14 days before birth was associated with a decrease in leukocyte (6.7%, 95% CI:-12.1,-1.0) and monocyte counts (11.6%, 95% CI:-19.6,-2.8). Maternal smoking during pregnancy was associated with significantly lower cord blood cell counts in multiple cell populations. Moreover, we observed sex differences regarding eosinophilic granulocytes and plasmacytoid dendritic cells. Finally, significantly increased numbers of cord blood cells were observed in infants exposed to perinatal stress. Cesarean section seems to play a significant role in Th1/Th2 balance.

**Conclusions:**

Our results suggest that all three: environmental, hereditary and perinatal factors play a significant role in the composition of cord blood cells at birth, and it is important to adjust for all of these factors in cord blood studies. In particular, perinatal circumstances seem to influence immune balance, which could have far reaching consequences in the development of immune mediated diseases.

## Introduction

It is known that the late prenatal and neonatal period represent a critical window of immune vulnerability and on-going immune maturation [1]. Cellular and soluble markers from infantile cord blood are used as risk biomarkers in many outcome studies investigating the origin of immune mediated disease during this early window of immune maturation. Special interest was paid to immune development in terms of allergy and tolerance. However, a multitude of environmental, hereditary, and perinatal factors can influence the composition of cord blood cells. During fetal life, the immune response is physiologically skewed towards a Type 2 helper T cell (Th2) response [2]. At birth, the infant’s immune system begins a process of re-calibration towards a Type 1 helper T cell (Th1) response [3, 4]. It is known that dendritic cells play an important role in the pathogenesis of asthma and allergic disease influencing Th1/Th2 balance [5], and that these cells are influenced by nitrogen dioxide (NO_2_),[6] particulate matter (PM) [7] and tobacco smoke in adults [8, 9].

It has been shown that *environmental factors*, such as prenatal ambient air pollution, have important effects on cord blood cells [10–13] and on cord blood cytokine profiles [14]. Maternal tobacco smoke affects leukocyte counts [15, 16], and reduces natural killer cell activity in umbilical cord blood [17]. *Hereditary fact*ors, both hereditary allergy risk [18] and sex [19, 20], have been reported to affect cord blood cells. Finally, the effect of *perinatal obstetric factors* on cord blood cells [19–22], and cytokines [23, 24] has been reported several times in the last decades.

Each of these studies was limited in that their focus was on subpopulations of cord blood cells [10–13, 21, 22], they investigated high exposure populations [10–12], they did not take into account all potentially known influencing factors in a healthy population [10–13, 18–22], nor they did assess individual exposure to air pollution [10–13]. Given that all cells within cord blood are likely affected by small and interacting effects of varying origin, an examination of cord blood composition accounting for all known confounders and influencing factors is needed. Thus, our aim was to investigate the relative impact of environmental, hereditary and perinatal factors on cord blood cells and on dendritic cells in particular, as master regulators of the Th1/Th2 balance. This investigation was carried out within a prospective birth cohort of healthy unselected term born neonates by way of a comprehensive multivariable statistical approach, using a sophisticated spatio-temporal exposure modeling of air pollutants.

## Materials and methods

### Ethics statement

The ethics committees of the regions of Bern and Basel approved the study and written informed consent of the parents was obtained at enrolment.

### Study design

Between April 1999 and July 2014, 295 subjects were enrolled from the prospective Basel-Bern Infant Lung Development Cohort (BILD). The study design, exclusion and inclusion criteria, and detailed questionnaires are reported in Fuchs et al. [25]. In brief, we investigated whether prenatal environmental exposures, hereditary and perinatal risk factors were associated with alterations in cord blood cell numbers in unselected middle European population of healthy new-borns prenatally recruited at the four major maternity hospitals and obstetric practices in the agglomeration of Bern, Switzerland. Exclusion criteria for this study were preterm delivery at <37 weeks of gestation, ethnicity other than European white, severe maternal health problems, maternal drug abuse other than nicotine, birth defects, major respiratory disease after birth, and/or significant perinatal disease.

### Outcome assessment

Cord blood was taken by venipuncture from the umbilical cord directly after birth and collected in sterile Ethylenediaminetetraacetic acid tubes. Blood analysis was performed within 12h after birth. Complete blood counts with correction for nucleated red blood cells were performed by the haematological laboratory of the University Hospital of Berne using a Cell-Dyn 3500R (Abbott, Baar, Switzerland). Dendritic cells as well as basophilic and eosinophilic granulocytes were, as previously described [16], flowcytometrically identified using a fluorescence-activated cell sorting scan (FACS-scan) (BD Bioscience, Franklin Lakes, NJ). The following monoclonal mouse anti-human antibodies, purchased from BD Bioscience, were used: anti-CD11c allocyanin (APC), anti-CD123 phycoerythrin (PE), lineage cocktail (lin 1) FITC (anti-CD3, CD14, CD16, CD19, CD20, and CD56), and anti-HLA-DR peridinin chlorophyll protein (PerCP). Mouse anti-human IgG2a APC, PerCP, FITC, and mouse anti-human IgG1 PE were used as isotype controls. Cells were identified as follows: Step 1) mononuclear cells were gated based on forward and side scatter signals; Step 2) cells where then selected within the gated population by four color-flowcytometry; myeloid dendritic cells: lin 1 negative, HLA-DR positive and CD11c bright; plasmacytoid dendritic cells: lin 1 negative, HLA-DR positive and CD123 bright; basophilic granulocytes: lin 1 negative, HLA-DR negative and CD123 positive; eosinophilic granulocytes: lin1 dim, HLA-DR dim, CD11c positive, CD123 dim.

Six children were excluded from further analysis due to technical errors. Of the 289 infants with good quality cord blood samples, in 261 we performed a differential leukocyte blood count, and 246 had a measurement of thrombocytes. In 253 children we were able to perform a FACS-scan determining basophilic granulocytes, eosinophilic granulocytes and dendritic cells. The different numbers in the FACS scan outcomes are due to an updated gating strategy for cord blood dendritic cells implemented after October 2006. Although all original FACS scan analyses (n = 253) were stored on long life compact discs for 51Infants (recruitment 1999–2002), the original data were no longer readable so that a reanalysis according to the new gating strategies was not possible and those measurements had to be excluded from final analysis.

### Exposure assessment

#### Environmental exposure

Maternal NO_2_ exposure was assessed at the residential addresses of study participants using a spatio-temporal model [26]. The model is based on traffic data, road network, land use, population, and an NO2 dispersion model to account for spatial variability; seasonality, meteorological conditions and a continuous running fixed air quality monitor to capture temporal variability. This method allowed for the estimation of mean NO_2_ levels at the address of each study participant in the third trimester, at days 30 and 14 prior to delivery. These particular time points were selected as they were shown to be important for lung development and as possibly crucial periods for the influence of air pollution on the immune system [12, 27]. Individual exposure to PM_10_ was assessed for the same timepoints, averaging the daily PM_10_ mean values from the background Swiss National Air Pollution Monitoring Network station Payerne, which is located within the study area. As a proxy for long term traffic-related pollution exposure, the distance from the mothers’ homes to the closest major road of ≥6 meters in width was calculated (GIS; ArcGIS, version 9, Environmental System Research Institute, Redlands, USA) and classified into two categories: 0–50 meters and ≥50 meters based on a previous study from our group [14]. Home addresses were geo-coded using the building registry of the Swiss Federal Statistical Office, and street information was obtained from the VECTOR25 map of the Swiss Federal Office of Topography (Wabern).

Maternal and paternal smoking habits during pregnancy were assessed by means of a standardized questionnaire. Maternal smoking habits were validated by chromatographic measured cotinine levels in the first urine of the newborns [25]. In one child the mother’s smoking history (nonsmoker) was contradicted by a high urine cotinine level (93ng/mL), and was therefore reclassified. Maternal passive smoking exposure was determined based on information about smoking exposure at home and at the workplace.

#### Hereditary factors

Several factors were assessed at enrolment based on a standardized questionnaire: sex, paternal atopic disease history, and maternal atopic disease history (allergic -rhinitis, -eczema, and -asthma), as described [25].

#### Perinatal factors

Information about perinatal circumstances were recorded by a responsible midwife at birth: parity, vaginal or cesarean delivery, meconium stained amniotic fluid, Apgar-Scores, umbilical vein pH, umbilical artery pH, pathological cardiotocography (CTG), premature rupture of the membranes, and gestational age.

### Statistical analysis

Cell counts were inspected for normality and, if necessary, were transformed before analysis (leukocytes, monocytes, lymphocytes, segmented neutrophils, basophils, eosinophils, pDCs, mDCs and pDC/mDC). Univariable, bivariable and multivariable linear regression models were applied to investigate the association between cord blood cell counts and known environmental, hereditary and perinatal risk factors. Covariates of concern were chosen *a priori* from the literature and included in the model. First we ran a univariable model for each risk factor against blood cells (simple model). Based on results from simple models and to minimize the multiple comparison issues, we focused on the pathological CTG as a main perinatal parameter representing stress during delivery. Subsequently, all cell counts were adjusted for known risk factors: sex, gestational age, birth order (classified as first born and subsequent born infants), mode of delivery, pathological CTG, maternal smoking during pregnancy, maternal atopy, season of birth (cosine term), the concentration of either PM_10_ or NO_2_ during the last trimester, the last 30 or 14 days preceding delivery (adjusted model). The cell subtypes measured by FACS were additionally controlled for the change in gating strategy (0 –measurements until October 2006, 1-after that date). We repeated the analysis restricting our sample size to participants without maternal smoking activity during pregnancy to assess the effect of maternal exposure to passive smoking on cord blood cells.

In the sensitivity analysis, we additionally adjusted the models with PM_10_ exposure for the residential distance to major roads.

Results are expressed as absolute difference in cell counts for untransformed outcomes and as a percent difference for log-transformed outcomes [100 × (exponentiated mean -1)] with 95% confidence interval (CI). Effect estimates for air pollution are presented per standardized increment of 10μg/m^3^. A p-value <0.05 was considered significant. All statistical analyses were performed using Stata version 11.2 (StataCorp., College Station, TX, USA).

## Results

A subgroup of 295 infants of the BILD cohort was included in this study. Anthropometric data, air pollution exposure data, and the distribution of possible risk factors are given in Table 1. Complete blood count analyses as well as FACS analysis results are given in Table 2.

**Table 1:** Population characteristics.

	Mean(±SD) / n(%)	n
Demographic Characteristics		
Gestational age, weeks	39.8 (±1.1)	278
Air pollution exposure		
*Outdoor*		
Mean PM_10_, last 14 days before delivery, μg/m^3^	18.4 (±8.0)	278
Mean PM_10_, last 30 days before delivery, μg/m^3^	19.1 (±7.1)	278
Mean PM_10_, third trimester of pregnancy, μg/m^3^	19.1 (±5.3)	278
Mean NO2, last 14 days before delivery, μg/m^3^	18.5 (±7.4)	265
Mean NO2, last 30 days before delivery, μg/m^3^	18.5 (±7.4)	265
Mean NO2, third trimester of pregnancy, μg/m^3^	18.5 (±7.2)	265
Distance to major roads, m	327 (±484)	276
*Indoor*		
Maternal smoking during pregnancy, yes	25 (9%)	278
Passive smoking during pregnancy, yes	53 (19.1%)	277
Hereditary Factors		
Sex, male	153 (55%)	278
Maternal atopy, yes	94 (33.8%)	278
Perinatal Factors		
Mode of delivery, cesarean section	42 (15.1%)	278
Pathological CTG, yes	27 (9.7%)	278
Meconium within amniotic fluid, yes	37 (13.4%)	277
Older siblings, yes	169 (60.8%)	278
Premature rupture of the fetal membranes, yes	16 (5.9%)	270

Abbreviations: PM_10_, Particulate matter <10 μm in diameter; NO_2_, nitrogen dioxide; CTG, Cardiotocogramm

**Table 2:** Cord blood cell counts and FACS analysis.

	Mean (±SD)	n
Total Leukocytes (x10^9^/l)	13.7 (±4.4)	289
Banded neutrophils (x10^9^/l)	0.8 (±0.7)	260
Segmented neutrophils (x10^9^/l)	5.9 (±2.7)	261
Monocytes (x10^9^/l)	1.3 (±0.6)	260
Lymphocytes (x10^9^/l)	4.9 (±1.9)	261
Eosinophilic granulocytes (x10^9^/l)	0.39 (±0.4)	253
Basophilic granulocytes (x10^9^/l)	0.05 (±0.04)	252
mDCs (x10^9^/l)	0.007 (±0.005)	199
pDCs (x10^9^/l)	0.010 (±0.008)	199
Thrombocytes (x10^9^/l)	283 (±66.4)	246

Abbreviations: mDC, myeloid dendritic cells; pDC, plasmacytoid dendritic cells

### Environmental exposure

Results for environmental exposures are summarized in Table 3. In general, the statistical associations were less significant in the simple models. Due to a high correlation between predicted NO_2_ values (Pearson correlation coefficient *r* = 0.91–0.99) (S1 Table) we observed similar effects of NO_2_ on cord blood cell counts at all studied time intervals. Given that the strongest effect of NO_2_ was mainly observed during the last 14 days before birth (S1 Fig), we are reporting results obtained from the models adjusted for this time period. PM_10_ and NO_2_ showed a moderate correlation (Pearson correlation coefficient *r* = 0.37–0.59, depending on the time period) (S1 Table).

**Table 3:** Simple^a^ and adjusted^b^ associations of cord blood cells with environmental risk factors.

	Maternal smoking during pregnancy				NO_2_ during the last 14 days before delivery^c^			
	Simple model		Adjusted model		Simple model		Adjusted model	
	β [95% CI]	p-value	β [95% CI]	p-value	β [95% CI]	p-value	β [95% CI]	p-value
**Leukocytes** ^d^	-17.5 [-27.9, -5.4]	0.006	-18.6 [-28.8, -7.0]	0.003	-4.9 [-9.8, 0.3]	0.064	-6.7 [-12.1, -1.0]	0.023
**Banded neutrophils** ^e^	-11.4 [-39.3, 29.3]	0.528	-9.3 [-37.8, 32.0]	0.609	-9.3 [-21.8, 5.1]	0.195	-15.5 [-28.6, 0.1]	0.052
**Segmented neutrophils** ^d^	-1.07 [-1.90, -0.24]	0.011	-1.17 [-2.05, -0.29]	0.009	-0.24 [-0.72, 0.24]	0.326	-0.43 [-0.98, 0.12]	0.128
**Monocytes** ^d^	-25.0 [-39.7, -6.7]	0.010	-24.6 [-39, -6.7]	0.009	-8.4 [-16.0–0.2]	0.046	-11.6 [-19.6, -2.8]	0.012
**Lymphocytes** ^d^	-24.3 [-37.3, -8.5]	0.004	-16.9 [-29.7; 2]	0.029	-1.7 [-8.2, 5.2]	0.618	-4 [-11; 3.4]	0.280
**Eosinophilic granulocytes** ^d^	-14.4 [-44.4, 31.6]	0.477	-15.0 [-44.9, 30.0]	0.463	-3.3 [-18.3, 14.3]	0.691	-2.9 [-20.7, 10.1]	0.777
**Basophilic granulocytes** ^d^	-38.0 [-56.6, -11.6]	0.009	-37.3 [-56.3, -11.2]	0.009	-0.7 [-13.7, 14.3]	0.922	-1.0 [-16.2, 16.8]	0.900
**pDCs** ^d^	-40.6 [-62.7, -5.5]	0.028	-42.9 [-63.3, -11.1]	0.013	-0.3 [-16.4, 19.0]	0.980	2.6 [-16.0, 25.2]	0.799
**mDCs** ^d^	-36.4 [-59.1, -1.2]	0.044	-36.6 [-59.6, -0.80]	0.046	-9.1 [-23.1, 7.5]	0.264	-11.3 [-27.6, 8.6]	0.245
**pDC/mDC** ^d^	-7.8 [-36.9, 34.8]	0.676	-10.7 [-38.5, 30.3]	0.555	7.8 [-6.6, 23.4]	0.300	14.0 [-4.0, 35.2]	0.133
**Thrombocytes** ^e^	-38.3 [-69.5, -7.00]	0.017	-45.9 [-77.0, -14.7]	0.004	6.0 [-6.5, 18.5]	0.344	9.65 [-4.46, 23.8]	0.179

Abbreviations: NO_2_, nitrogen dioxide; β, coefficient; CI, confidence interval; mDC, myeloid dendritic cell; pDC, plasmacytoid dendritic cell

^a^ basophilic granulocytes, eosinophilic granulocytes, pDCs, mDCs, and the pDCs/mDCs ratio were additionally adjusted for the change in gating strategy; for other cells, univariable associations are presented.

^b^ adjusted for sex, gestational age, birth order, gestational age, mode of delivery, CTG, maternal smoking during pregnancy, maternal atopy, season of birth and 14 days average of NO_2_.

^c^ Effect estimates for NO_2_ are presented per 10 μg/m^3^ NO_2_ increase

^d^ Results are expressed as percent difference

^e^ Results are expressed as a difference in absolute cell counts

Multivariable analysis revealed significant negative associations between exposure to NO_2_ and leukocyte and monocyte counts (β = -6.7, 95% CI:-12.1,-1.0, and β = -11.6, 95% CI:-19.6,-2.8, respectively) (Table 3 and S1 Fig). There was no association between PM_10_ exposure and any of the cord blood cells (data not shown).

Cord blood cell numbers, except those for banded neutrophils and eosinophilic granulocytes, were significantly decreased in neonates with maternal smoking exposure during pregnancy (Table 3). When we restricted our sample size to neonates without maternal smoking exposure during pregnancy, we found no evidence for an association between maternal passive smoking exposure and cord blood cell counts (data not shown).

### Hereditary risk factors

Eosinophilic granulocyte counts and pDCs were significantly higher in boys compared to girls in adjusted models (S2 Table). We did not observe a significant difference in cord blood cell numbers between neonates with and without maternal atopy or maternal asthma (data not shown).

### Perinatal risk factors

**Fig 1:**
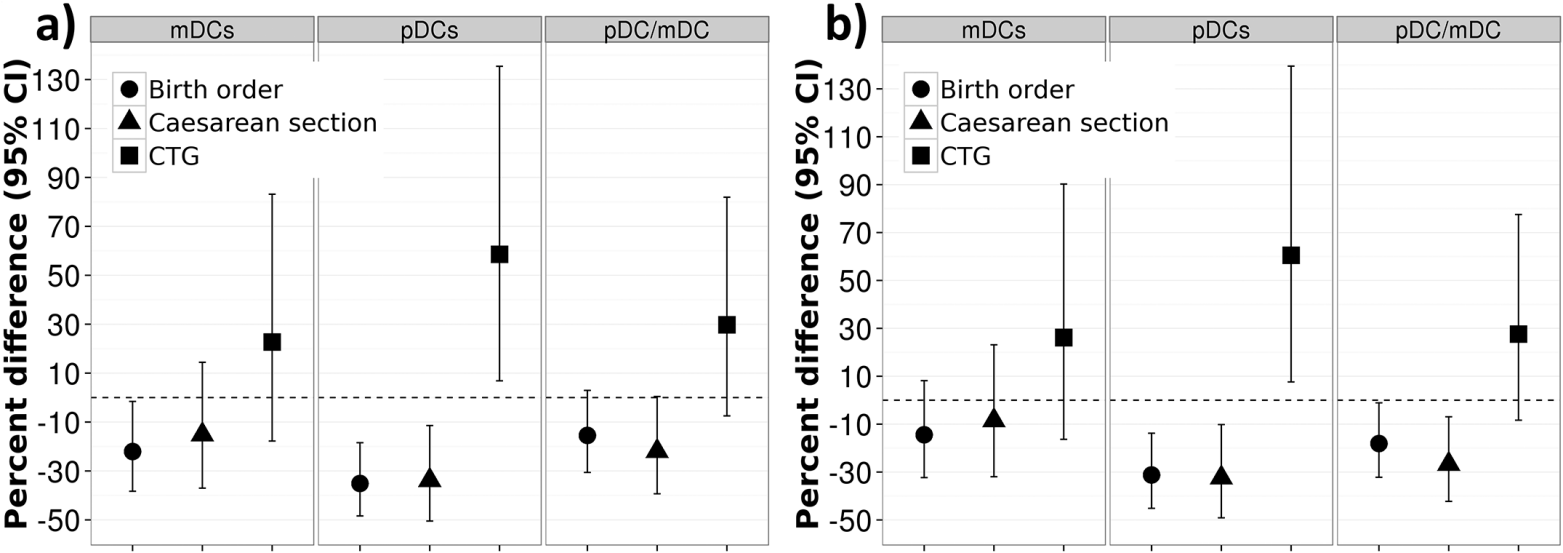
a+b. Adjusted^a^ effect of perinatal stress factors on mDCs, pDCs, and pDCs/ mDCs ratio in multivariable models using a) NO_2_ and b) PM_10_ exposure during last 14 days before the birth. ^a^ adjusted for sex, gestational age, birth order, gestational age, mode of delivery, CTG, maternal smoking during pregnancy, maternal atopy, change in the gating strategy, and season of birth.

In the simple models, the second and subsequent births (*birth order*) were associated with decreased monocyte, basophilic granulocyte and pDC counts in neonates (Table 4). Associations were stable after adjustment for other risk factors. In the multivariable model, including exposure to NO_2_, the effect of birth order was also seen for mDC counts (β = -22.1; 95% CI:-38.3,-1.56). However, we did not observe any significant association between birth order and mDC counts after adjustment for PM_10_ exposure (Fig 1).

**Table 4:** Simple^a^ and adjusted^b^ associations of cord blood cells with perinatal risk factors.

	Birth order				Mode of delivery				CTG			
	Simple model		Adjusted model		Simple model		Adjusted model		Simple model		Adjusted model	
	β [95% CI]	p-value	β [95% CI]	p-value	β [95% CI]	p-value	β [95% CI]	p-value	β [95% CI]	p-value	β [95% CI]	p-value
**Leukocytes** ^c^	-6.5 [-13.7, 1.3]	0.102	-5.5 [-12.8, 2.31]	0.161	-10.6 [-20.0, -0.1]	0.048	-11.9 [-21.0, -1.7]	0.024	18.2 [3.9, 34.5]	0.011	16.6 [2.7, 32.4]	0.018
**Banded neutrophils** ^d^	-19.9 [-35.9; 1.0]	0.051	-17.6 [-34.3, 3.3]	0.094	-8.3 [-32.5, 24.5]	0.578	-9.4 [-33.5, 23.3]	0.526	40.1 [-3.8, 4.1]	0.078	39.1 [-4.6, 102;9]	0.086
**Segmented neutrophils** ^c^	-0.4 [-1.2, 0.3]	0.282	-0.32 [-1.1, 0.4]	0.409	-0.7 [-1.6, 0.2]	0.129	-0.76 [-1.7, 0.18]	0.113	-0.1 [-1.3, 1.0]	0.804	-0.2 [-1.3, 1.0]	0.754
**Monocytes** ^c^	-13.6 [-24.1, -1.6]	0.028	-13.5 [-23.9, -1.74]	0.026	-7.5 [-22.7, 10.8]	0.399	-9.6 [-24.2, 7.9]	0.264	40.7 [12.9, 75.6]	0.003	33.9 [7.8, 66.5]	0.009
**Lymphocytes** ^c^	-1.3 [-10.9, 9.3]	0.801	-1.7 [-11.1, 8.6]	0.733	-4.6 [-16.8, 9.4]	0.499	-5.6 [-17.8, 8.3]	0.409	46.9 [24.4, 73.5]	<0.001	43.2 [20.8, 69.7]	<0.001
**Eosinophilic granulocytes** ^c^	-9.7 [-28.8, 14.5]	0.398	-14.6 [-33.3, 9.3]	0.193	-18.4 [-41.4, 13.6]	0.226	-22.7 [-44.8, 8.1]	0.221	19.1 [-19.7, 76.8]	0.383	8.7 [-27.2, 62.4]	0.899
**Basophilic granulocytes** ^c^	-21.8 [-35.8, -4.8]	0.015	-21.3 [-35.7, -3.7]	0.020	-20.0 [-40.0, 4.1]	0.094	-25.4 [-43.3, -1.9]	0.036	42.4 [2.7, 97.6]	0.034	30.7 [-5.7, 81.4]	0.108
**pDCs** ^c^	-33.5 [-46.2, -16.2]	0.001	-35.1 [-48.4, -18.4]	<0.001	-30.3 [-48.6, -5.5]	0.021	-33.8 [-50.5, -11.4]	0.006	77.6 [18.4, 166.4]	0.006	58.6 [6.9, 135.4]	0.022
**mDCs** ^c^	-19.8 [-36.0, 0.4]	0.054	-22.1 [-38.3, -1.56]	0.036	-11.7 [-34.3, 18.8]	0.409	-15.1 [-37.0, 14.4]	0.280	32.8 [-10.0, 96.1]	0.152	22.7 [-17.7, 83.1]	0.314
**pDC/mDC** ^c^	-16.5 [-31.1, 1.1]	0.065	-15.5 [-30.6, 3.0]	0.094	-21.8 [-39.1; 0.4]	0.054	-21.9 [-39.3, 0.4]	0.054	32.1 [-5.1, 84.0]	0.099	29.7 [-7.5, 81.9]	0.130
**Thrombocytes** ^d^	11.6 [-6.4, 29.7]	0.206	10.9 [-7.3, 29.1]	0.238	-18.9 [-42.8; 5.0]	0.120	-19.7 [-43.5, 4.2]	0.105	-36.8 [-65.5, -8.2]	0.012	-41.1 [-69.9, -12.2]	0.005

Abbreviations: CTG, Cardiotocogramm; β, coefficient; CI, confidence interval; mDC, myeloid dendritic cell; pDC, plasmacytoid dendritic cell

^a^ basophilic granulocytes, eosinophilic granulocytes, pDCs, mDCs, and the pDCs/mDCs ratio were additionally adjusted for the change in gating strategy; for other cells univariable associations are presented.

^b^ adjusted for sex, gestational age, birth order, gestational age, mode of delivery, CTG, maternal smoking during pregnancy, maternal atopy, season of birth, and 14 days average of NO_2_

^c^ Results are expressed as percent difference

^d^ Results are expressed as a difference in absolute cell counts

Neonates delivered via *C-section* had lower cord blood cell numbers than neonates delivered vaginally (Table 4). The effect of mode of delivery was stronger in adjusted models. Significant associations were found for leukocyte and basophilic granulocyte counts and pDCs. The pDC/mDC ratio was decreased in neonates delivered via C-section with a borderline significance level (β = -21.9; 95% CI:-39.3,04), due to a more pronounced reduction in pDC than in mDC counts (β = -33.8; 95% CI:-50.5,-11.4 and β = -15.1; 95% CI:-37.0,14.4, respectively) (Table 4 and Fig 1a). However, in the multivariable model, which accounted for PM_10_ exposure, we observed a significant association between the pDC/mDC ratio and the delivery mode (β = -26.7; 95% CI:-42.3, -6.9) (Fig 1b).

*Prenatal Stress* expressed by pathological CTG tended to increase cord blood cell numbers except for thrombocytes, which showed a decrease (Table 4). Adjustment for other confounders attenuated the magnitude of the associations, but most of them remained significant. Cell numbers associated with pathological CTG include: leukocytes, monocytes, lymphocytes, pDCs, and thrombocytes.

### Sensitivity analysis

There was no evidence for an association between distance to major roads and cord blood cell counts (data not shown). Adjusted models suggested statistically significant dose-response trends between the number of cigarettes smoked per day and a decrease in leukocyte-, lymphocyte-, monocyte-, basophilic granulocyte-, pDC-, and thrombocyte-counts (data not shown). Sensitivity analyses revealed only small trend differences between 14 infants (out of 36) delivered by emergency C-section and 22 infants born by elective caesarean section. These borderline trends were not reported due to lack of statistical power.

## Discussion

Our results showed that even low level prenatal *environmental exposure* to NO_2_ and maternal smoking, but not to PM_10_, are associated with a significant decrease in multiple cord blood cell types. We showed a pronounced effect of mode of delivery on total leukocyte counts, basophilic granulocytes, and specifically pDC, with significantly lower levels of those cell populations in cesarean-born children. Cesarean-born children showed a borderline significant decrease in the pDC/mDC ratio, suggesting an immunological imbalance in cord blood. *Sex specific effects* were weak, only eosinophilic granulocytes and pDCs were significantly higher in boys compared to girls in adjusted models.

### Comparison with previous air pollution exposure studies

Three studies from a larger project out of the Czech Republic reported a distinct effect of air pollution on cord blood cells. In one study, PM exposure during pregnancy was found to be associated with higher natural killer cell fractions in newborns [11]. A subsequent study found a decrease in T-lymphocyte fractions and an increase in B-lymphocyte fractions in cord blood by higher concentrations of PM_2.5_ 14 days before birth [12]. Finally, a gestational-stage specific significant effect of PM_2.5_ on cord blood lymphocyte distribution was reported [10]. A French research group showed analogous results with increasing PM_10_ and NO_2_ exposure levels three months prior to and during pregnancy [13].

The concentrations of pollutants to which the mothers in our study were exposed are comparable to the French study [13] but differ widely from those in the Czech Republic [10–12]. In contrast to our study, both groups assessed the effect of outdoor pollution on cord blood cells without an individually modelled PM or NO_2_ exposure. Furthermore, outcomes were a percent of lymphocyte subpopulations which makes it difficult to compare their findings with the absolute cord blood cell values in our study. Moreover, differences in the findings from the Czech studies, in comparison to our study, may also be related to differences in particle size.

Several toxicology studies have described the effect of short-term exposure to NO_2_, presenting mixed results and hypotheses. Two independent studies showed a significant decrease of peripheral blood cells after NO_2_ exposure [28, 29]. 80–90% of inspired NO_2_ is absorbed in humans as nitrite, metabolized and then excreted in the urine [30]. Steenhof et al. [29] concluded that nitrite, as well as secondary oxidation products, may induce the observed systemic effects of NO_2_ exposure on peripheral blood cells in adults. Translating those findings to our study population we presume that nitrite and secondary oxidation products circulating in the mother’s blood can pass the placental barrier and generate the observed effects in the offspring’s blood.

Regarding the effect of maternal smoking on the cord blood cells of the infants, we were able to confirm the results of a previous publication [16]. One of our novel findings was the demonstration of a significant decrease in cord blood pDC, and in thrombocyte numbers, in infants of smoking mothers.

The effects of acute and chronic smoking on DCs are highly complex. A study in adult smokers showed *in vivo* a strong increase in mDC in bronchoalveolar lavage fluid, and a subsequent decrease in mDC in blood shortly after the onset of active smoking [8]. On the other hand, in chronic smokers, increased absolute DC values and an altered pDC/mDC ratio were found, compared to healthy controls [31]. The authors postulate that smoking may directly alter the immune response. As is well known, pDC and mDC are related to Th1 and Th2 immune responses, respectively. Thus, the hypothesis of tobacco induced immune modulation is also supported by other studies showing that in utero and postnatal exposure to environmental tobacco smoke skews the immune response towards a Th-2 phenotype [4, 9]. Despite the fact that smoking induced a significant decrease in cord blood dendritic cells in our study, the pDC/mDC ratio was not significantly polarized. We hypothesize that this finding may be affected by the relatively low dose tobacco exposure in participating mothers (<20 cigarettes/day) of our cohort. Smoking affects platelet activation [32]. The literature is, however, controversial about the effect of smoking on platelet counts in peripheral blood. The observed decrease of platelet counts in our population could possibly be explained through a higher aggregation under perinatal stress conditions of the pre-activated platelets.

### Effects of hereditary and perinatal factors

There remains a lack in literature on *hereditary influences* on cord blood cells [18]. To our knowledge, no study to date has analyzed the influence of maternal atopy or asthma on absolute values of cord blood cells in offspring. Given our sample size, we were unable to detect any significant difference in cord blood cell levels in relation to maternal atopy or asthma. Sex differences in multiple aspects of immune development and immune response have been previously published [19, 20, 33]. As a novel finding, our study showed significantly higher pDCs in boys.

The effect of *perinatal factors* on cord blood cellular composition [19–22, 24] and on cytokines [23, 24] has been investigated in the past. Our results are in line with previous studies showing significantly lower total leukocyte numbers in children born via C-section compared to vaginal delivery [19, 20, 24], and higher numbers of cord blood cells in infants exposed to higher perinatal stress [21]. As a novel finding, we describe a borderline significant decrease in the pDC/mDC ratio in cesarean-born, compared to vaginally born, children. This suggests a potential selective effect of mode of delivery on pDC. Since dendritic cells play an important role in immune regulation, and influence the Th1/Th2 balance in the development of asthma and allergies, [5] further studies are needed to explore the potential modifying role of C-sections on DCs, the Th1/Th2 balance in early infancy, and the association of these findings to the subsequent risk of chronic immune disorders (e.g. asthma) later in life [34].

### Methodological considerations

This is the first study, to our knowledge, investigating the effect of individually modeled outdoor air pollution, hereditary, and perinatal factors on absolute cord blood cell counts; and, in particular, on dendritic cells in cord blood.

A major strength of our study is the comprehensive approach accounting for the influence of multiple factors (environmental, hereditary, and perinatal) on cord blood cell counts. Our study distinguishes itself from previous studies investigating the effect of prenatal NO_2_ exposure on immunologic parameters of offspring due to our use of a time-space regression model to assess individual exposure for relevant time periods.

A major limitation of our study was that blood samples were assessed subsequently. In October 2006, due to the updated standards in the gating of DC in FACS, a new gating strategy was applied. Although all the available original FACS analyses prior to this date were re-gated according to the new gating strategies, a trend in higher dendritic cell values before the new gating strategy persisted. We adjusted for this in our final statistical models. PM_10_ exposure was not individually sampled; rather we used a simpler model to estimate levels from a background sampling station. This provides a good picture regarding temporal variability, which is known to be much more relevant for PM [35], but is weak in representing spatial variability. Due to the fact that total leukocyte counts consist of the sum of analyzed white blood cell subsets, some reported associations between total leukocytes and the different exposures might be influenced by specific subsets. On the other hand, considering that there is a common progenitor cell for all white blood cells, the association between multiple cell subsets and different exposures could be traced back to an effect on a common progenitor cell.

## Conclusion

In conclusion, our results suggest that environmental, hereditary and perinatal factors play a significant role in the composition of cord blood cells at birth. Research studies involving cord blood cell counts need to be adjusted for all of these factors. Furthermore, this dataset may serve as a well characterized normative dataset for a Central-European population. In particular, avoidable factors such as NO_2_, even at low levels, and tobacco smoke seem to play an important role in cord blood cell composition. Moreover, perinatal circumstances seem to influence the immune balance, which could be important to explain of the association between C-sections and the subsequent risk of immune mediated diseases later in life [34].

## Supporting information

S1 Table Pearson correlation coefficients across all time intervals. (DOCX)

S2 Table Simple and adjusted associations of cord blood cells with sex. (DOCX)

S1 Fig Adjusted effect of NO_2_ on leukocytes, monocytes and banded neutrophils. (DOCX)
