# Supplementary material for: Response of cord blood cells to environmental, hereditary and perinatal factors: A prospective birth cohort study
Source: PLoS One. 2018 Jul 6;13(7):e0200236. doi: 10.1371/journal.pone.0200236 (PMC6034853; doi:10.1371/journal.pone.0200236)
Supplement: S1 Table — (DOCX) [file pone.0200236.s001.docx]

**S1 Table: Pearson correlation coefficients across all time intervals.**

|  | **NO_2_** | | |  | **PM_10_** | | |
| --- | --- | --- | --- | --- | --- | --- | --- |
|  | **14 days** | **30 days** | **3rd trimester** |  | **14 days** | **30 days** | **3rd trimester** |
| **NO_2_** | | | | | | | |
| **14 days** | 1 |  |  |  |  |  |  |
| **30 days** | 0.99 | 1 |  |  |  |  |  |
| **3rd trimester** | 0.91 | 0.94 | 1 |  |  |  |  |
| **PM_10_** | | | | | | | |
| **14 days** | 0.48 | 0.47 | 0.37 |  | 1 |  |  |
| **30 days** | 0.55 | 0.58 | 0.52 |  | 0.79 | 1 |  |
| **3rd trimester** | 0.46 | 0.51 | 0.59 |  | 0.52 | 0.75 | 1 |

Abbreviations: PM_10_, Particulate matter <10 µm in diameter; NO_2_, nitrogen dioxide
