# Supplementary material for: Response of cord blood cells to environmental, hereditary and perinatal factors: A prospective birth cohort study
Source: PLoS One. 2018 Jul 6;13(7):e0200236. doi: 10.1371/journal.pone.0200236 (PMC6034853; doi:10.1371/journal.pone.0200236)
Supplement: S2 Table — (DOCX) [file pone.0200236.s002.docx]

**S2 Table: Simple and adjusted associations of cord blood cells with sex.**

|  |  | |  | | | |
| --- | --- | --- | --- | --- | --- | --- |
|  | **Sex** | | | | | |
|  | **Simple model** | | |  | **Adjusted model** | |
|  | **β**  **[95% CI]** | **p-value** | |  | **β**  **[95% CI]** | **p-value** |
| **Leukocytes ^d^** | -5.1  [-12.3, 2.7] | 0.196 | |  | -3.3  [-10.5, 4.5] | 0.400 |
| **Banded neutrophils ^e^** | -21.9  [-37.2, -2.8] | 0.027 | |  | -15.8  [-33.3, 6.3] | 0.147 |
| **Segmented neutrophils ^d^** | -0.9  [-1.6, -0.2] | 0.012 | |  | -0.7  [-1.4, 0.01] | 0.053 |
| **Monocytes ^d^** | -1.6  [-13.5, 11.9] | 0.805 | |  | 1.7  [0.9, 1.2] | 0.785 |
| **Lymphocytes ^d^** | 4.2  [-5.7, 15.1] | 0.418 | |  | 1.4  [-8, 11.8] | 0.780 |
| **Eosinophilic granulocytes^d^** | 32.5  [5.1, 67.1] | 0.018 | |  | 36.7  [7.4, 1.7] | 0.011 |
| **Basophilic granulocytes ^d^** | 10.3  [-9.4, 34.2] | 0.326 | |  | 15.6  [-4.8, 41.1] | 0.141 |
| **pDCs ^d^** | 18.3  [-6.0, 49.1] | 0.151 | |  | 29.2  [3.2, 61.7] | 0.025 |
| **mDCs ^d^** | 5.2  [-15.7, 31.3] | 0.650 | |  | 7.8  [-14.4, 35.7] | 0.521 |
| **pDC/mDC ^d^** | 9.0  [-8.7, 31.6] | 0.366 | |  | 16.3  [-4.3, 41.2] | 0.128 |
| **Thrombocytes ^e^** | -4.7  [-22.3, 12.9] | 0.598 | |  | -0.7  [-18.3, 16.9] | 0.989 |

Abbreviations: β, coefficient; CI, confidence interval; mDC, myeloid dendritic cells; pDC, plasmacytoid dendritic cells

^a^ basophilic granulocytes, eosinophilic granulocytes, pDCs, mDCs , and the pDCs / mDCs ratio were additionally adjusted for the change in gating strategy; for other cells univariable associations are presented.

^b^ adjusted for sex, gestational age, birth order, gestational age, mode of delivery, CTG, maternal smoking during pregnancy, maternal atopy, season of birth, and 14 days average of NO_2_.

^c^ Sex is coded as Female (0) and Male (1). The results represent the change in cells in males compared to females.

^d^ Results are expressed as percent difference

^e^ Results are expressed as a difference in absolute cell counts
