# Supplementary material for: Response of cord blood cells to environmental, hereditary and perinatal factors: A prospective birth cohort study
Source: PLoS One. 2018 Jul 6;13(7):e0200236. doi: 10.1371/journal.pone.0200236 (PMC6034853; doi:10.1371/journal.pone.0200236)
Supplement: S1 Fig — (DOCX) [file pone.0200236.s003.docx]

**S1 Fig: Adjusted effect of NO_2_ on leukocytes, monocytes and banded neutrophils**

Adjusted^a^ effect of NO_2_ on leukocytes, monocytes and banded neutrophils

^a^ adjusted for sex, gestational age, birth order, gestational age, mode of delivery, CTG, maternal smoking during pregnancy, maternal atopy, and season of birth

Abbreviations: CI, confidence interval; NO_2_, nitrogen dioxide

**
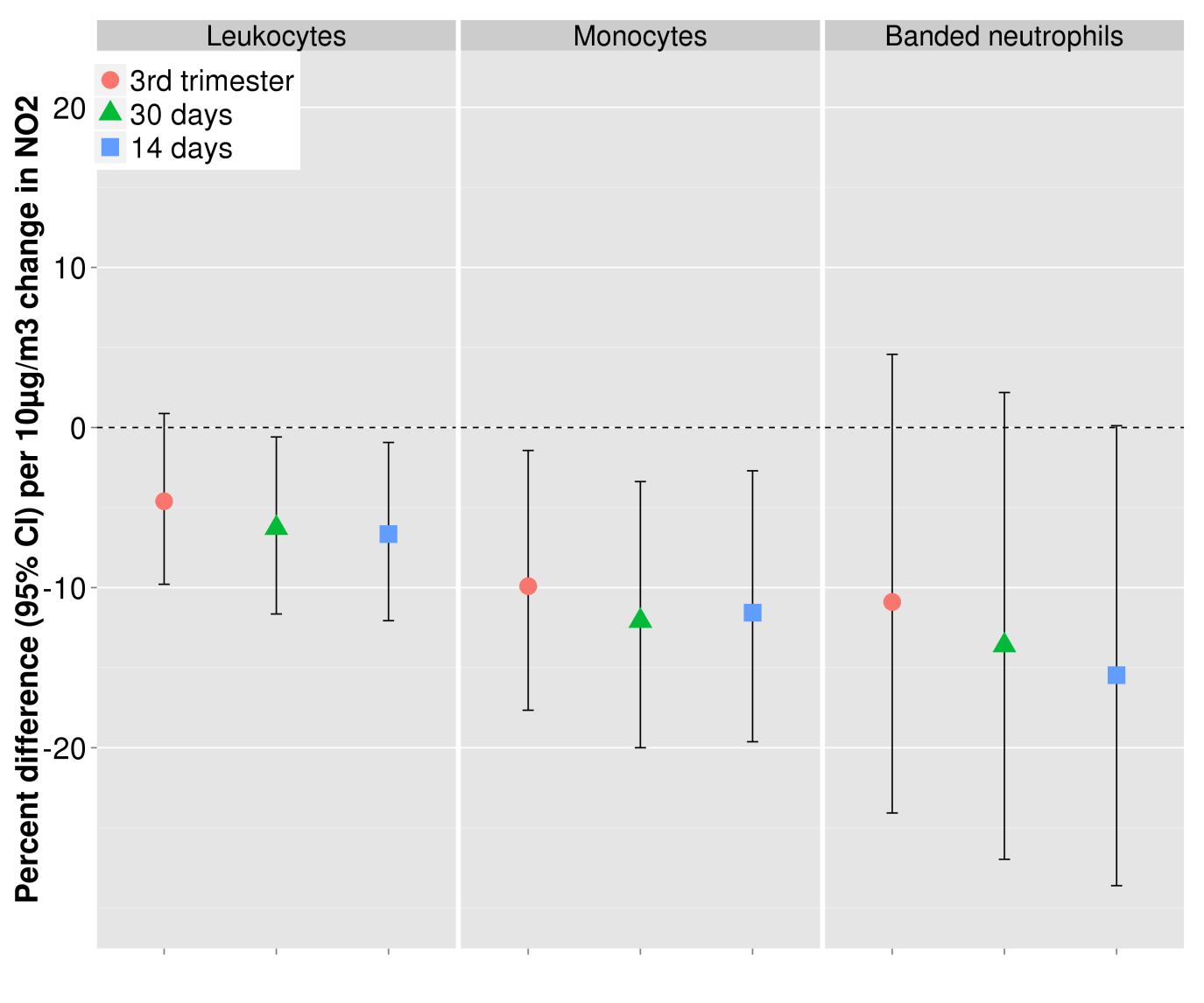
**
